# The Analgesic Efficacy and Safety of Intramuscular Hydromorphone Versus Butorphanol for Acute Pain in the Emergency Department: A Randomized Trial

**DOI:** 10.1155/prm/4981039

**Published:** 2026-06-16

**Authors:** Yongping Huang, Xiaojing Peng, Bihua Zhang, Li Luo, Yufang Sun, Hang Dai, Jimei Zhang, Jia Yin, Shiqiang Xiong, Tao Xiang

**Affiliations:** ^1^ Department of Emergency, The Third People’s Hospital of Chengdu, Chengdu, 610031, Sichuan, China, scu.edu.cn; ^2^ Department of Radiology, The Shenzhen Hospital of Southern Medical University, Shenzhen, 518101, Guangdong, China; ^3^ Department of Cardiology, The Third People’s Hospital of Chengdu, Chengdu, 610031, Sichuan, China, scu.edu.cn

**Keywords:** acute pain, butorphanol, emergency department, hydromorphone

## Abstract

**Background:**

Acute pain represents a distressing encounter for most patients in the emergency department (ED), profoundly impacting their quality of life. Nonsteroidal anti‐inflammatory drugs (NSAIDs) and morphine represent the most widely utilized analgesics; however, their clinical application in acute pain management is limited by their delayed onset of pharmacological action or significant risk of addiction. This study aimed to evaluate and compare the analgesic efficacy and safety of intramuscular hydromorphone and butorphanol in patients with acute pain in the ED.

**Methods:**

This randomized controlled clinical trial randomly divided 199 patients with moderate to severe acute pain into two groups in a 1:1 ratio, receiving intramuscular injections of 1 mg of hydromorphone or butorphanol, respectively. Primary outcomes included pain intensity changes measured by the numerical rating scale (NRS), while secondary outcomes encompassed supplemental analgesic requirements, vital sign alterations, adverse event incidence, and patients’ destination.

**Results:**

Comparative analysis revealed that hydromorphone administration resulted in significantly greater NRS score reductions compared to butorphanol, with superior pain relief rates observed at both 1 (56% vs. 44%, *p* = 0.013) and 2 h (69% vs. 60%, *p* = 0.002) intervals, and a greater proportion of apparent relief at 2 h (88% vs. 69%, *p* = 0.002), particularly in cases of severe pain (78% vs. 56%, *p* = 0.001). The hydromorphone group demonstrated significantly lower supplemental analgesic requirements at 2 h postadministration (0.0% vs. 6.1%, *p* = 0.031). There was no significant difference in the incidence of adverse reactions, the final destination, and the effects on heart rate, respiration, and oxygen saturation between the two groups. Common adverse reactions included dizziness or headache, nausea or vomiting, and injection pain.

**Conclusion:**

Both hydromorphone and butorphanol were effective and safe in alleviating moderate to severe acute pain in the ED. However, hydromorphone exhibited superior analgesic effectiveness and a more rapid onset of action compared to butorphanol, particularly in cases of severe pain.

**Trial Registration:** Chinese Registry of Clinical Trial: ChiCTR2400088376

## 1. Introduction

Pain is recognized as the “fifth vital sign” [1–3], causing serious physical and psychological damage to patients, and is one of the major causes of disability [4]. Previous studies have confirmed that the administration of early analgesia does not hinder disease diagnosis; rather, it can effectively protect organ function, alleviate ischemic and hypoxic injury, and improve patient comfort and compliance with medical recommendations [3, 5].

Currently, analgesic drugs include nonsteroidal anti‐inflammatory drugs (NSAIDs), acetaminophen, and opioids. Among these, both domestic and international guidelines recommend opioids as an option for the treatment of moderate to severe pain (numerical rating scale [NRS] ≥ 4) [3, 4, 6]. Morphine, the most classic and widely used opioid analgesic, is increasingly associated with high rates of abuse and addiction, making the development of alternative drugs an urgent necessity [7–9]. Clinical studies have indicated that hydromorphone and butorphanol exhibit higher bioavailability, a more rapid onset of action, and enhanced analgesic effect compared to morphine [10–13]. At equivalent doses, their analgesic potency is approximately 5–7 times and 3–7 times that of morphine, respectively [9, 14–16]. Furthermore, the incidence of adverse reactions is comparable to or lower than that of morphine [10–13, 17–19], making them reasonable choices for the management of postoperative or cancer‐related pain [11, 12, 16, 17].

Statistically, more than 50.0% of emergency department (ED) visits are primarily for pain complaints, and 60.0%–80.0% of ED patients experience pain [1–3, 20–23]. Patients presenting to the ED with pain typically exhibit an acute onset and severe intensity, often accompanied by an ambiguous diagnosis. The selection of analgesic regimens for these patients differs significantly from that of chronic or postoperative pain. Although several clinical studies suggest that hydromorphone may offer effective analgesia for acute pain in the ED [24–26], robust clinical evidence regarding the safety, efficacy, and timeliness of hydromorphone and butorphanol in acute pain management remains limited. Therefore, this study aimed to evaluate and compare the analgesic potential of these two agents in patients experiencing acute pain in the ED.

## 2. Methods

### 2.1. Study Design and Settings

This is a prospective, randomized, single‐blind, parallel group, single‐center trial conducted from September 1, 2023, to January 31, 2024, in the ED of the Third People’s Hospital of Chengdu, Chengdu, China. Reporting was in accordance with the CONSORT statement. This study was approved by the Ethics Review Committee of the Third People’s Hospital of Chengdu (Ethical approval number: 2023‐S‐153) and obtained written informed consent from all participating subjects. All procedures and protocols were conducted in accordance with the Declaration of Helsinki.

### 2.2. Participants

Trained emergency physicians conducted a rigorous screening of patients in accordance with the inclusion and exclusion criteria established by the investigators. The inclusion criteria comprised the following: (1) patients experiencing acute moderate to severe pain (NRS ≥ 4) requiring opioid analgesic treatment, (2) adult patients (aged ≥ 18 years), and (3) obtained informed consent from the patient. The exclusion criteria included the following: (1) pregnant or lactating women; (2) individuals with a known allergy to opioids, including hydromorphone or butorphanol; (3) chronic opioid users or those with a history of opioid addiction; (4) presence of medical conditions that may affect the metabolism of opioid analgesics (severe hepatic or renal insufficiency); (5) systolic blood pressure less than 90 mmHg, heart rate (HR) less than 50 beats per minute (bpm), or oxygen saturation less than 90% on room air; (6) intoxication by alcohol or other drugs; (7) presence of symptoms of respiratory depression, suffering from acute or severe bronchial asthma; (8) presence or risk of progression to gastrointestinal obstruction, especially paralytic intestinal obstruction; (9) loss of hearing or vision, or any other condition that may significantly hinder data collection for the study; (10) communication deficits (e.g., psychiatric or neurological disorders or sedation); (11) opioid use within the previous 24 h; (12) concurrent use of medications that may interact with the study medications; and (13) concurrently participated in any clinical trial.

### 2.3. Randomization

Randomization was conducted by an emergency physician scanning a QR code, which randomly assigned patients in a 1:1 ratio to receive either hydromorphone or butorphanol. The emergency physician prescribed the randomly generated medication for the patient and hand‐delivered it to the specialized nurse, who administered the injection. The specialized nurse retrieved the medication from the dedicated medication cabinet to administer injections to patients. Patients and their legal guardians remained blinded to the specific medication administered until the end of the study observation period.

### 2.4. Intervention

The hydromorphone and butorphanol groups received intramuscular injections of 1 mg hydromorphone hydrochloride (preinfused) (Yichang Human Well Pharmaceutical Co., Ltd, Hubei, China) and 1 mg butorphanol tartrate (Jiangsu Hengrui Pharmaceutical Co., Ltd, Jiangsu, China), respectively. Two hours later, participants were queried regarding their need for additional analgesic medication. If the answer was yes, an additional 1 mg of the same medication was administered.

### 2.5. Outcomes

Pain intensity was measured by the NRS, which is represented by an integer from 0 to 10, with the side of 0 as no pain and 10 as the worst pain. The rating scale was administered via verbal inquiries with patients. The amount of NRS change was indicated as the premedication NRS (NRS_1_) subtracted from the postmedication NRS (NRS_2_), and the rate of NRS change relative to NRS_1_ was indicated as the rate of pain relief [27]. Pain relief rate ≥ 50.0% was considered apparent relief [28–30].

Pain relief rate = (NRS_1_−NRS_2_)/NRS_1_ ∗ 100%.

The primary outcome was the pain relief rate at 0.5, 1, and 2 h after administration and the percentage of apparent relief. The secondary outcomes included whether additional analgesics were still needed after 2 h of medication; changes in participants’ HR, respiratory rate (RR), mean arterial pressure (MAP), and pulse oxygen saturation (SpO_2_) at different times; and the occurrence of medication or injection related adverse effects such as nausea, vomiting, dizziness, respiratory depression, and needle‐sickness, as well as the final destination of the participants.

### 2.6. Statistics

Based on the following parameters: two‐sided alpha of 0.05, one‐sided alpha of 0.10, SD of 2.8 units for the 0–10 NRS scale, and a minimum clinically significant difference of 1.3 units [24, 25]. We calculated that a minimum of 196 subjects would be required for this study. To account for potential losses during follow‐up, we ultimately included 200 subjects.

Statistical analysis was performed using SPSS 25.0. Normally distributed data were expressed as mean ± standard deviation (x ± s), and group comparisons were made using two independent sample *t*‐test. Non‐normally distributed data were expressed as median (interquartile range) and statistically analyzed using nonparametric tests. Categorical data were expressed as frequencies, component ratios, or percentages and were subjected to chi‐square tests. A *p* value < 0.05 was considered to be statistically significant.

## 3. Results

### 3.1. Patient Characteristics

**FIGURE 1:**
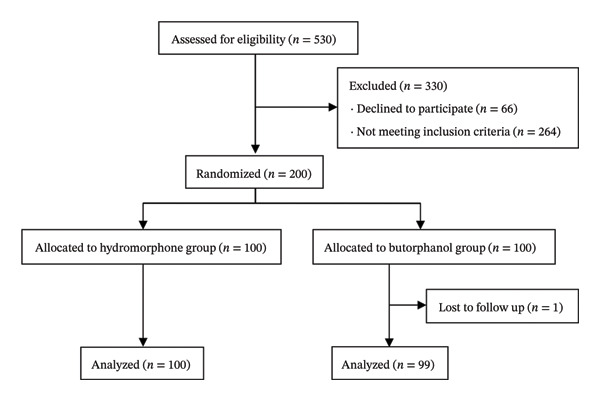
Consolidated standards of reporting trials diagram.

A total of 530 patients with acute pain were screened during the study period; 200 patients who met the inclusion and exclusion criteria were randomized. Following randomization, 100 patients were allocated to the hydromorphone group and 99 to the butorphanol group, with one participant lost to follow‐up (Figure 1). Baseline demographic and clinical characteristics were comparable between the two groups (Table 1). The cohort predominantly comprised male patients (63.3%), with the most frequently reported pain locations being abdominal (60.3%) and lumbar (30.2%) regions. Pain severity revealed that 28 patients (14.1%) experienced moderate pain (NRS 4–6) [3], while the majority, 171 patients (85.9%), presented with severe pain (NRS ≥ 7) [3] (Table 1).

**TABLE 1:** Baseline characteristics.

	**Hydromorphone (*n* = 100)**	**Butorphanol (*n* = 99)**	***p* value**

Gender, *n* (%)			0.62
Male	65 (65.0)	61 (61.6)	
Female	35 (35.0)	38 (38.4)	
Age (years), mean ± SD	43.5 ± 15.5	44.7 ± 16.4	0.578
Weight (kg), mean ± SD	65.9 ± 9.9	66.6 ± 10.5	0.644
NRS, median [IQR]	8 [7–9]	8 [7–8]	0.06
Pain location, *n* (%)			0.385
Abdomen	60 (60.0)	60 (60.6)	
Waist	33 (33.0)	27 (27.3)	
Others	7 (7.0)	12 (12.1)	
Pain intensity, *n* (%)			0.399
Moderate	12 (12.0)	16 (16.2)	
Severe	88 (88.0)	83 (83.8)	
Vital signs, mean ± SD			
HR (bpm)	79.6 ± 12.5	82.4 ± 15.8	0.170
MAP (mmHg)	102.3 ± 14.1	101.7 ± 14.4	0.772
RR (bpm)	19.8 ± 1.6	19.7 ± 1.4	0.807
SpO_2_ (%)	98.4 ± 1.0	98.2 ± 1.3	0.230

*Note:* Values are *n* (%).

Abbreviations: bpm, beats per minute; HR, heart rate; IQR, interquartile range; MAP, mean arterial pressure; NRS, numerical rating scale; RR, respiratory rate; SD, standard deviation; SpO_2_, pulse oxygen saturation.

### 3.2. Efficacy

**FIGURE 2:**
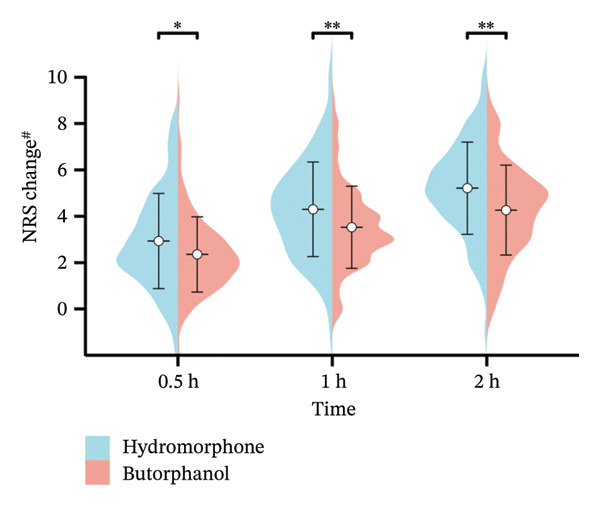
Amount of change in NRS in the hydromorphone and butorphanol groups. Note: ^#^premedication NRS subtracted from postmedication NRS at different times. ^∗^ *p* < 0.05 and ^∗∗^ *p* < 0.01. Abbreviation: NRS: numerical rating scale.

There was no significant difference in initial NRS between the two groups (8 [7–9] vs. 8 [7, 8], *p* > 0.05) (Table 1). The amount of change in NRS was significantly higher in the hydromorphone group than in the butorphanol group at 0.5, 1, and 2 h after medication (*p* < 0.05) (Figure 2), and the rate of pain relief in the hydromorphone group was also significantly higher than in the butorphanol group at 1 and 2 h (*p* < 0.05) (Table 2). In addition, the percentage of apparent pain relief after 2 h of medication was higher in the hydromorphone group than in the butorphanol group (88.0% vs. 69.7%, *p* < 0.01) (Table 2).

**TABLE 2:** Comparison of the changes in NRS, additional analgesics, and patient destinations.

	**Hydromorphone (*n* = 100)**	**Butorphanol (*n* = 99)**	***p* value**

Pain relief rate, %			
0.5 h	38 [22–50]	29 [17–43]	0.081
1 h	56 [38–71]	44 [33–57]	0.013^∗^
2 h	69 [56–80]	60 [43–71]	0.002^∗∗^
Apparent relief, *n* (%)	88 (88.0)	69 (69.7)	0.002^∗∗^
Additional analgesic, *n* (%)	0 (0.0)	6 (6.1)	0.014^∗^
Destination, *n* (%)			0.778
Return home	71 (71.0)	67 (67.7)	
Observed in an emergency	19 (19.0)	19 (19.2)	
Admission	10 (10.0)	13 (13.1)	

*Note:* Values are *n* (%).

Abbreviation: NRS, numerical rating scale.

^∗^ *p* < 0.05.

^∗∗^ *p* < 0.01.

**FIGURE 3:**
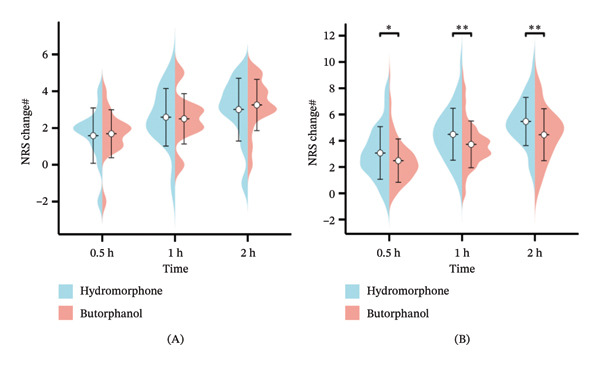
Stratified analysis of the amount of change in NRS in the hydromorphone and butorphanol groups with different pain intensity. Note: ^#^Premedication NRS subtracted from postmedication NRS at different times. ^∗^ *p* < 0.05 and ^∗∗^ *p* < 0.01. Abbreviations: moderate pain (A) and severe pain (B); NRS: numerical rating scale.

Subsequently, we conducted a more in‐depth stratified analysis to investigate the differences in analgesic effects between the two groups with different pain intensities (Table 3 and Figure 3). There were no significant differences in pain relief rates; the amount of changes in NRS at 0.5, 1, and 2 h; and the percentage of apparent relief at 2 h between the two groups in patients with moderate pain (*p* > 0.05). In contrast, among patients with severe pain, the amount of NRS change at 0.5 h, 1 h, and 2 h; the rate of pain relief at 1 h and 2 h; and the percentage of apparent pain relief at 2 h after medication in the hydromorphone group were significantly higher than those in the butorphanol group (*p* < 0.05).

**TABLE 3:** Stratified analysis of the effects of hydromorphone and butorphanol with moderate and severe pain.

	**Moderate pain**			**Severe pain**		
	**Hydromorphone (*n* = 12)**	**Butorphanol (*n* = 16)**	***p* value**	**Hydromorphone (*n* = 88)**	**Butorphanol (*n* = 83)**	***p* value**
Pain relief rate, %						
0.5 h	33 [21–40]	33 [17–40]	0.925	38 [22–50]	29 [14–43]	0.145
1 h	45 [33–67]	45 [33–50]	0.464	56 [39–71]	44 [33–57]	0.006^∗∗^
2 h	60 [50–83]	60 [50–67]	0.851	71 [56–79]	57 [43–71]	0.001^∗∗^
Apparent relief, *n* (%)	10 (83.3)	13 (81.3)	1	78 (88.6)	56 (67.5)	0.001^∗∗^
Adverse reaction, *n* (%)			0.791			0.835
Yes	2 (16.7)	1 (6.3)		17 (19.3)	15 (18.1)	
No	10 (83.3)	15 (93.8)		71 (80.7)	68 (81.9)	
Additional analgesics, *n* (%)				0 (0.0)	6 (7.2)	0.031^∗^

*Note:* Values are *n* (%).

^∗^ *p* < 0.05.

^∗∗^ *p* < 0.01.

We further analyzed and compared the need for additional medication during the treatment process between the two groups. The results indicated that none of the patients in the hydromorphone group requested supplemental analgesic medication within the initial 2 h after treatment. In contrast, six patients (6.1%) in the butorphanol group with severe pain required an additional 1 mg of analgesic medication during the same time frame, demonstrating a statistically significant intergroup difference (*p* < 0.05) (Tables 2 and 3). Furthermore, an evaluation of patient’s final destination following treatment showed no significant differences between the two groups (Table 2).

### 3.3. Safety

**FIGURE 4:**
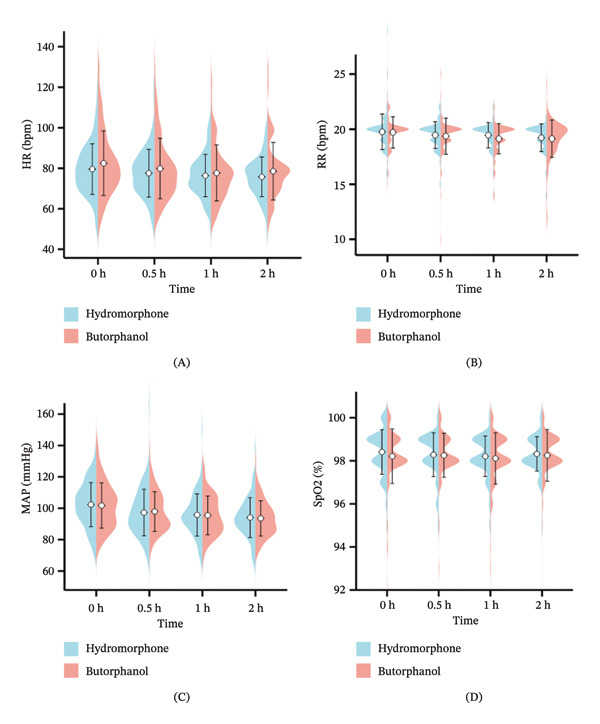
Changes in vital signs at different times in the hydromorphone and butorphanol groups (A–D). Abbreviations: heart rate (A), respiratory rate (B), mean arterial pressure (C), and pulse oxygen saturation (D); bpm: beats per minute.

The overall incidence of adverse reactions was 19.0% in the hydromorphone group and 16.2% in the butorphanol group, with moderate pain (16.7% vs. 6.3%) and severe pain (19.3% vs. 18.1%) (Tables 3 and 4). However, statistical analysis revealed that these differences were not significant (*p* > 0.05). Commonly reported adverse reactions in both groups included dizziness or headache (64.0% vs. 55.0%), nausea or vomiting (20.0% vs. 35.0%), injection site pain (12.0% vs. 10.0%), and hypotension (4.0% vs. 0.0%), with no instances of respiratory depression observed in either group (Table 4). Furthermore, no significant differences were detected between the two groups in terms of their effects on HR, RR, MAP, or SpO_2_ (Figure 4).

**TABLE 4:** Incidence of adverse reactions.

	**Hydromorphone (*n* = 100)**	**Butorphanol (*n* = 99)**	***p* value**

Adverse reaction, *n* (%)			0.599
Yes	19 (19.0)	16 (16.2)	
No	81 (81.0)	83 (83.8)	
Types of adverse reactions, *n* (%)			
Dizziness or headaches	16 (64.0)	11 (55.0)	
Nausea or vomiting	5 (20.0)	7 (35.0)	
Injection site pain	3 (12.0)	2 (10.0)	
Hypotension	1 (4.0)	0 (0.0)	

*Note:* Values are *n* (%).

## 4. Discussion

This randomized controlled trial investigated the efficacy of emergency analgesia for acute pain management, comparing intramuscular administration of hydromorphone and butorphanol. Both agents demonstrated significant pain relief. However, hydromorphone exhibited a more rapid onset of action and superior analgesic potency relative to butorphanol, particularly in cases of severe pain. The safety profiles of both analgesics were comparable, with no significant differences in patient tolerance observed. Notably, hydromorphone administration was associated with a reduction in total opioid consumption.

Previous studies have suggested that hydromorphone possesses favorable early analgesic properties [10, 11, 24–26], but others have reported no significant advantage in analgesic efficacy or safety when compared to alternative opioids (e.g., morphine and oxycodone) [17–19, 31, 32]. Furthermore, a systematic review encompassing 24 studies concluded that hydromorphone does not exhibit superior analgesic efficacy or a reduced incidence of adverse effects relative to morphine [15]. Subsequent analysis revealed that these studies primarily focused on cancer‐related pain and postoperative pain management. As the treatment of such conditions often requires sustained high plasma concentrations of analgesics to maintain prolonged pain relief, hydromorphone, as a short‐acting opioid analgesic, may not fully demonstrate its potential advantages and therapeutic efficacy in these contexts. In contrast, short‐acting opioid analgesics are particularly advantageous for the treatment of acute pain, especially in the ED. However, when searching the data, we found a paucity of clinical studies examining the use of hydromorphone for acute pain management in the ED. This may be attributed to the challenges associated with the initial diagnosis of pain in ED, as the administration of analgesics prior to a definitive diagnosis, especially in patients with acute abdominal pain, is conventionally believed to potentially delay accurate diagnosis [33, 34]. At the same time, the high rate of patient loss to follow‐up further complicates clinical research in the ED. Our study found that approximately 70.0% of patients were discharged home after emergency treatment, while only 10.0% required hospital admission for further treatment, with incomplete data often available for nonadmitted patients.

Multiple clinical trials investigating acute pain management in ED have demonstrated the superior analgesic efficacy of hydromorphone compared to acetaminophen, morphine, or lidocaine [10, 24–26]. We also found that both hydromorphone and butorphanol significantly relieved patients’ pain, with intramuscular injection of 1 mg hydromorphone achieving 66.7% pain relief within two hours, surpassing the 60.0% efficacy observed with butorphanol. Furthermore, 88.0% of patients in the hydromorphone group reported apparent pain relief, compared to 69.7% in the butorphanol group. However, stratified analysis revealed that this advantage was only in patients presenting with severe pain. Notably, the limited number of moderate pain cases included in this study may affect the accuracy of the findings. Therefore, it is necessary to validate these results in a larger sample of patients with moderate pain. In addition, in the early stages of medication, the amount of changes in NRS was more favorable for hydromorphone than butorphanol, suggesting enhanced analgesic potency during the initial treatment period.

Chang AK et al. [35] reported that 55.0% of patients achieved pain relief with only 1 mg of hydromorphone injections, while 45.0% required additional doses exceeding 1 mg. In contrast, our findings indicate that 88.0% of patients experienced significant pain relief following a single 1 mg hydromorphone injection without requiring supplemental analgesics. This difference may be attributed to methodological differences in assessment timing: Chang et al. [35] evaluated the need for additional analgesics at 30‐min intervals, whereas our study employed a 2‐h assessment. Previous studies have found that intravenous hydromorphone reaches peak plasma concentrations at approximately 20 min, achieves maximal analgesic efficacy by 30 min, has a terminal half‐life of 2–3 h, and maintains therapeutic effects for up to 2 h [36, 37]. Accordingly, we selected the 2 h mark for assessment, a timing that also follows the recommendations in the package insert for hydromorphone hydrochloride injection, which suggests administering the medication every 2 to 3 h as needed [38]. This time point selected can reduce overall analgesic consumption, given that research confirms that excessive or repeated use of opioid or nonopioid analgesics can increase the risk of adverse reactions and addiction [9, 35, 39], potentially leading to analgesic shortages in critical clinical situations [7, 8]. It must be noted, however, that in accordance with pharmacological recommendations, our study involved asking patients after 2 h regarding the need for additional analgesics, unless an earlier request had been made. In real‐world clinical practice, individuals experiencing pain often require optimal relief sooner, and a 2‐h interval may inadvertently prolong discomfort for some. Consequently, the timing of rescue analgesia should be guided by the patient’s pain status and a careful assessment of the benefit–risk balance related to potential adverse events. This aspect warrants further investigation.

In clinical practice, the administration of opioid analgesics necessitates heightened vigilance regarding potential severe adverse effects, particularly respiratory depression, which poses a significant risk in critically ill patients and may increase mortality rates [13]. While some previous studies have reported that hydromorphone can cause significant reductions in SpO_2_, HR, or blood pressure [35], our research revealed that neither hydromorphone nor butorphanol significantly change in HR, RR, or SpO_2_, with the exception of a single case of hypotension attributed to hydromorphone. In addition, the most frequently observed adverse reactions to both hydromorphone and butorphanol were dizziness or nausea, consistent with prior reports [11, 26, 35]. These results support the conclusion that hydromorphone is a safe option for emergency analgesia.

## 5. Limitations

Limitations of this study mainly include the following: First, the study was a single‐center clinical trial, and hydromorphone or butorphanol may not be available at all healthcare facilities. The reliability and stability of its findings require further verification through large‐scale, multicenter clinical studies. To address this limitation, our team is conducting a multicenter clinical study. Second, this study used a single‐blind design, which may introduce selection bias. While participants were blinded to group assignment to ensure objective outcomes, physicians and nurses were not blinded due to the regulatory requirement that psychiatric and anesthetic medications must be prescribed by licensed physicians, as well as the center’s actual operational circumstances. Third, the study was restricted to a 2‐h follow‐up period, during which over 25.0% of patients continued to experience moderate to severe pain. The persistence of the observed clinical benefits with prolonged medication duration remains unclear, potentially introducing bias into the interpretation of the results.

## 6. Conclusion

In the ED, the application of hydromorphone or butorphanol can significantly alleviate patients’ acute moderate to severe pain, thus improving their healthcare experience. Both agents demonstrate comparable rates of adverse events. However, comparative analysis reveals that hydromorphone exhibits superior analgesic efficacy and a more rapid onset of action relative to butorphanol. Consequently, hydromorphone exhibits greater clinical utility in the management of acute pain in the ED, especially in patients with severe pain.

## Author Contributions

Yongping Huang, Xiaojing Peng, and Bihua Zhang contributed equally to this work and share the first authorship. They analyzed and summarized the data and drafted the manuscript. Li Luo and Hang Dai contributed to the study conception and design. Data collection was completed by Yufang Sun, Jimei Zhang, and Jia Yin. Tao Xiang and Shiqiang Xiong revised the manuscript in detail.

## Funding

No funding was received for this research.

## Disclosure

This manuscript has been presented as a preprint on “Research Square” (https://doi.org/10.21203/rs.3.rs-6831108/v1) [40]. All authors have read and agreed to the published version of the manuscript.

## Conflicts of Interest

The authors declare no conflicts of interest.

## Data Availability

The datasets used or analyzed during the current study are available from the corresponding author on reasonable request.
